# Evidence of cancer: a systematic review of metabolomics in extracellular vesicles for cancer biomarker detection

**DOI:** 10.1007/s11306-025-02386-1

**Published:** 2025-12-16

**Authors:** Megan Mayhew, Oliver Megram, Simmie Roshan, Marshall J. Smith, Samuel J. White, Philippe B. Wilson, John A. Hunt, Luigi De Girolamo, Victoria James, Elena Hunter

**Affiliations:** 1 School of Science and Technology, Nottingham Trent University, Nottingham, UK; 2 Present Address: Laboratory of Chemical Physics, National Institute of Diabetes and Digestive and Kidney Diseases, National Institute of Health, Bethesda, MD 20892 USA; 3 Present Address: Centre for Applied Innovation, York St John University, York, UK; 4 Present Address: York St John University, York, UK; 5 Medical Technologies Innovation Facility, Nottingham Trent University, Nottingham, UK; 6 Biodiscovery Institute, School of Veterinary Medicine and Science, University of Nottingham, Nottingham, UK

**Keywords:** Metabolomics, Extracellular vesicles, Biomarkers, Cancer, Metabolites

## Abstract

**Background:**

The early detection of cancer remains a critical challenge in clinical oncology, with significant implications for patient survival rates and treatment outcomes. Research focus has shifted to developing minimally invasive diagnostic approaches for cancer detection and prognosis, such as metabolomic analysis of biological fluids and tumour-derived components, including extracellular vesicles (EVs). EVs carry molecular cargo, including metabolites, that reflect the pathophysiological state of their cell of origin. Analysis and characterisation of these metabolites may offer novel insights into cancer biology and facilitate the identification of potential biomarkers.

**Aim of review:**

This review systematically examines existing literature on the metabolomic analysis of EVs in the context of cancer to obtain a deeper understanding of potential metabolite biomarkers associated with cancer.

**Key scientific concepts of review:**

A comprehensive search of PubMed, Scopus, and Web of Science was conducted using a defined strategy to identify studies analysing EV-derived metabolites in cancer. Twelve eligible studies were included, collectively reporting 1,602 identified metabolites across various cancer types, sample sources, EV isolation methods, and metabolomic techniques. Of these, 333 metabolites were reported to be differentially regulated in EVs derived from patients with cancer, or conditioned medium from cancerous cell lines and their respective healthy controls. The review highlights the potential of EV metabolomics to detect cancer biomarkers but also underscores methodological variability as a major limitation. Differences in isolation and analytical techniques likely contribute to inconsistent findings, emphasising the need for standardised protocols in future research.

**Supplementary Information:**

The online version contains supplementary material available at 10.1007/s11306-025-02386-1.

## Introduction

Detecting cancer early and accurately continues to pose a significant challenge in modern healthcare, with only 54% of cases in England being diagnosed at stage 1 or 2 in 2021 (NHS Digital, 2023). When detected, diagnosed, and treated early, the likelihood of survival increases significantly for almost all types of cancer due to improved treatment options (Crosby et al., 2022).Screening techniques often focus on specific cancer types, such as mammograms, pap smears, and prostate-specific antigen tests. These techniques are typically only offered to predetermined target populations, which may result in missed diagnoses (Weller & Campbell, 2009). Furthermore, these screening techniques have individual limitations, including sensitivity, specificity, and patient participation in screening initiatives (Barry, 2001; Ferreira et al., 2021; Grimm et al., 2022; Miglioretti et al., 2016). Implementing more accessible screening initiatives in both high-risk and general populations could contribute to the early detection of cancer, providing more opportunities for timely treatment and ultimately leading to improved mortality rates (Sung et al., 2021).Tumour biopsy is the gold standard, providing a histological evaluation of a tumour, including type, staging and hormone receptor presence (Ma et al., 2024; Vaidyanathan et al., 2018). However, tumour biopsies can only capture a single time point and site (Perakis & Speicher, 2017; Rodríguez et al., 2021).Therefore, they are limited in their ability to provide a comprehensive understanding of the dynamic landscape of a tumour due to genetic instability and intra-tumour heterogeneity (Campbell et al., 2010; Gerlinger et al., 2012; Perakis & Speicher, 2017). Moreover, the invasive nature of tumour biopsies exposes patients to various risks, including the potential for tumour spread, injury to the surrounding tissue, severe bleeding, and infection (Alieva et al., 2018). Consequently, there is a clinical need for less invasive procedures for early detection and diagnosis. These approaches can enhance patient adherence to screening and monitoring programmes whilst simultaneously minimising the potential risks and complications associated with invasive diagnostic procedures (Febbo et al., 2024). One such emerging technique is liquid biopsy. Conducted on readily accessible biological fluids such as peripheral blood, liquid biopsies involve biomarker identification as a minimally invasive, early-stage cancer screening and longitudinal monitoring method (Russano et al., 2020).

A metabolomics approach is one possible way to analyse liquid biopsies (Wang et al., 2023).This technique comprehensively analyses endogenous metabolites, less than 1.5 kDa in size, present in a biological system (Clarke & Haselden, 2008; Rankin et al., 2014; Wishart et al., 2007).These metabolites include a range of molecular species such as carbohydrates, organic acids, lipids, fatty acids, and amino acids (AAs) (Palomo et al., 2014; Rankin et al., 2014). Metabolomics approaches require the use of analytical techniques such as nuclear magnetic resonance (NMR) spectroscopy, gas chromatography-mass spectrometry (GC–MS), or liquid chromatography-mass spectrometry (LC–MS) to analyse liquid biopsies for the presence of tumour derivatives and cancer biomarkers (Palomo et al., 2014). The metabolic profile output by such techniques provides a snapshot of the metabolic state of a sample, such metabolic phenotypes can differentiate disease states (Schiliro & Firestein, 2021). Metabolic profiling for cancer detection is possible due to the dysregulated metabolism of cancerous cells, driven by rapid proliferation, which often results in distinct metabolic phenotypes reflected in cell derivatives, including intercellular metabolites, extracellular metabolites, and extracellular vesicles (EVs) (Schiliro & Firestein, 2021).

**Fig. 1:**
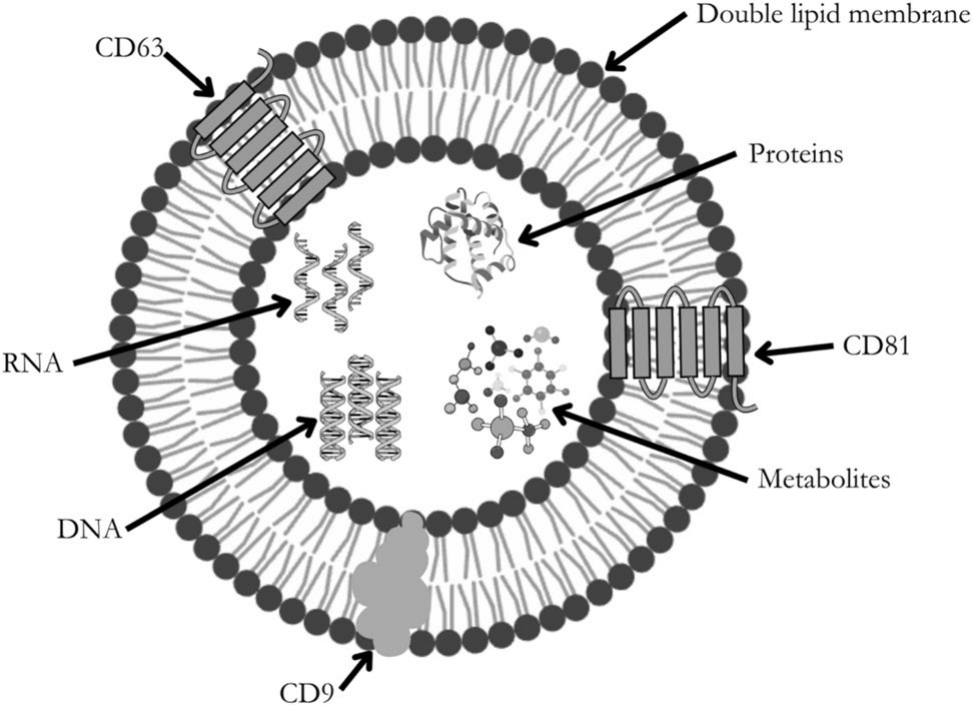
The structure and content of extracellular vesicles

The term EVs refers to non-replicating nanoparticles synthesised and released by a cell (Théry et al., 2018; Witwer et al., 2013). While EVs are generated and released by all cells, cancer cells, particularly those from higher-grade and more aggressive tumours, have been shown to consistently generate larger quantities and sizes of EVs (D’Souza-Schorey Crislyn & Clancy, 2012; Robles-Flores Editor, 2014). EVs are differentiated into three main subtypes: exosomes, microvesicles (MVs), and apoptotic bodies. Each EV subtype plays a distinct role within cells and contributes uniquely to cancer progression and metastasis. In tumours, exosomes play a crucial role in the angiogenic and extracellular matrix management and modification of the tumour microenvironment to regulate tumour growth and metastasis (Dai et al., 2020; Kalluri & LeBleu, 2020). MVs are responsible for initiation of angiogenesis and apoptosis in target cells in tumours (Lu et al., 2017). Subtypes of MVs, such as oncosomes and tumour-derived MVs, are exclusively produced by cancer cells and often contain oncogenic proteins indicative of their cell of origin (D’Souza-Schorey Crislyn & Clancy, 2012; Rak, 2010; Robles-Flores Editor, 2014). Identifying these subtypes may help differentiate cancerous phenotypes from healthy controls (D’Souza-Schorey Crislyn & Clancy, 2012; Rak, 2010; Robles-Flores Editor, 2014). These vesicles, found in all biofluids, are enclosed by a lipid bilayer and carry a cell-type-specific cargo including RNA, DNA, proteins, and metabolites as shown in Fig. 1 (Liang et al., 2021; Yáñez-Mó et al., 2015). One of the primary purposes of EV cargo is to help facilitate the EV's involvement in physiological and pathological functions for homeostatic cell maintenance and disease progression, including cancer metastasis (Kalluri & LeBleu, 2020; Kim et al., 2020; Palomo et al., 2014; Perakis & Speicher, 2017). However, as the composition of this cargo mirrors the molecular and metabolic characteristics of the cell of origin at the point of EV biogenesis, its analysis is a valuable tool for assessing the physiological state of an organism (Palomo et al., 2014). Therefore, analysis of EV cargo could indicate the presence of a disease, disease-related alterations, and cancer-specific metabolic signatures within a sample (Kalluri & LeBleu, 2020; Kim et al., 2020; Palomo et al., 2014; Perakis & Speicher, 2017).

Current research into EV cargo has focused on the critical role of nucleic acids and proteins within EVs. However, there is limited research on the metabolites present in EVs, even though their metabolomic profiles could potentially reveal cancerous phenotypes (Rak, 2010). The presence of EVs in all biofluids, coupled with their ability to cross the blood–brain barrier, further advances their applications in diagnosis and longitudinal disease monitoring via liquid biopsy (Banks et al., 2020; Dardet et al., 2022; Kalluri & LeBleu, 2020). Consequently, the metabolomic analysis of EV cargo presents an innovative and non-invasive strategy for identifying cancer biomarkers within biological samples. Table 1 summarises some of the key advantages and challenges associated with using EVs in metabolomic studies, highlighting factors such as specificity, stability, accessibility, functional insights, biomarker potential, and opportunities for multi-omics integration. This article systematically reviews current research into the metabolome of EVs to obtain a deeper understanding of potential biomarkers associated with cancer.

**Table 1:** Advantages and disadvantages of using EVs in metabolomic studies

Aspect	Advantages	Challenges
Specificity	The composition of metabolites present within an EV mirrors the metabolic characteristic of the cell of origin. Therefore, EVs have potential use in assessing if parent cells are cancerous	Biological fluids contain heterogeneous populations of EVs. This heterogeneity presents a challenge for metabolomic studies, as metabolites detected in bulk EV isolates may represent a mixture from diverse sources, making it difficult to attribute specific metabolic signatures to cancerous cells
Stability	The metabolite contents of an EV are protected from degradation by the lipid bilayer membrane which forms part of the EV structure	EV isolation and storage can affect metabolite integrity. Shear forces from filtration and mechanical damage from freeze–thaw cycles can lead to cargo leakage
Non-invasiveness	EVs can be isolated from minimally invasive to collect biofluids such as blood, urine and saliva	EVs are present in low concentrations in these biofluids so may require large sample volumes for metabolomic analysis
Accessibility	EVs are present in all biological fluids and can cross the blood brain barrier	There is a lack of universally standardised protocols for the isolation and characterisation of EVs which can affect reproducibility and comparability across studies
Multi-omics integration	EVs also carry proteins, RNA, and lipids enabling comprehensive omics analysis	There is a lack of standardised methods for isolation, characterisation, and analysis making comparison and integration challenging

## Methods

### Search strategy

A systematic review was conducted to identify published articles that reported the metabolomic analysis of EVs as a diagnostic biomarker for any cancer in any species (up to the 18th of February 2024). The review utilised a defined search strategy (outlined in Table 2) and employed the electronic databases PubMed, Scopus, and Web of Science.

**Table 2:** Search terms used for each database

Database	Keywords	Results
Scopus	TITLE-ABS-KEY ( ( ( “extracellular vesicle”) OR ( exosome) OR ( microvesicle)) AND ( ( metabolite) OR ( metabolomic) OR ( metabolome) OR ( metabonomic)) AND ( ( cancer) OR ( tumour)))	(n = 1483)
Web of Science	(((“extracellular vesicle”) OR (exosome) OR (microvesicle)) AND ((metabolite) OR (metabolomic) OR (metabolome) OR (metabonomic)) AND ((cancer) OR (tumour)))	(n = 187)
PubMed	(((“extracellular vesicle”) OR (exosome) OR (microvesicle)) AND ((metabolite) OR (metabolomic) OR (metabolome) OR (metabonomic)) AND ((cancer) OR (tumour)))	(n = 453)

For this review, research papers were screened against the following inclusion criteria: the selected studies were original articles with full-text literature, published in English and included the following topics: EVs, cancer diagnostics, and metabolomics. From the database searches, each article's title, year of publication, authors, and abstracts were exported to Zotero v6.0.31, where duplicates were removed (Zotero, 2024). The remaining articles were imported into Rayyan, where screening was conducted based on the inclusion and exclusion criteria (Ouzzani et al., 2016). Initially, the title and abstract were screened, and studies were excluded if they were conference proceedings, case reports, opinion articles, review articles, letters, grey literature, or articles that did not contain keywords such as cancer, metabolomics, and EVs. Potential inclusions from this stage underwent full-text screening, where authors (MM, OM, SR) determined eligibility for inclusion. During the full-text screening, articles were excluded for the following reasons: (1) the article did not compare EVs obtained from the conditioned medium of cancerous cells with non-cancerous cells, nor did it compare EVs isolated from the biofluids of patients with cancer and controls without cancer; (2) the metabolomic analysis was not completed on the isolated EVs; (3) the article only analysed one metabolite or focused only on lipidomics.

### Data extraction

A data extraction form was completed for each of the included studies. It contained the authors’ names, year of publication, sample type, cancer type, sample populations, EV isolation technique, EV confirmation technique/s, metabolite extraction technique, employed metabolomic technique, metabolite identification method, and metabolites identified. Supplementary Information (SI Table 1) includes the extracted data.

## Results

**Fig. 2:**
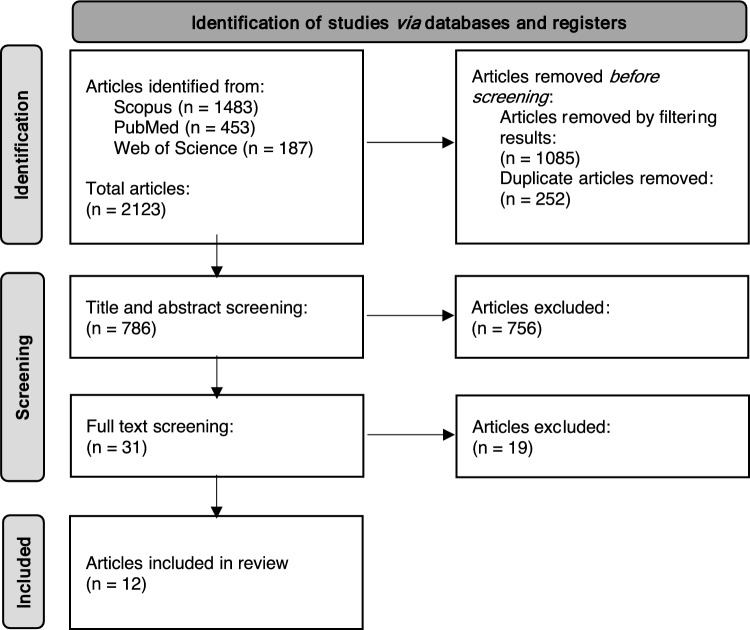
PRISMA Flow diagram of the article selection process and the number of articles included or excluded at each stage for this systematic review

As outlined in Fig. 2, 2123 articles were identified in Scopus, PubMed, and Web of Science using keywords and MeSH terms shown in Table 1. Before the screening stage, 252 duplicates were removed, and 1085 articles were removed through filtering. Of the 786 remaining articles, title and abstract screening removed 756 articles, the remaining 31 full-text articles were reviewed, and 12 were included in the final analysis as depicted in the flow diagram (Fig. 2). The selected studies were published between 2016 and 2023. They included colorectal cancer (CRC), endometrioid adenocarcinoma, glioblastoma, lung cancer including non-small cell lung cancer (NSCLC), cutaneous T-cell lymphoma (CTCL), oesophageal squamous cell carcinoma (OSCC), pancreatic cancer, and prostate cancer (PCa) including castration-resistant prostate cancer (CRPC) studies with some studies analysing more than one cancer type.

### Characteristics of the included studies

The studies included in this review exhibit a diverse range of sample characteristics, including cancer type, biofluid sources, and sample sizes. Table 3 summarises these sample characteristics for each study. Sample sizes ranged from 6 to 102 participants in the studies that used human biological fluids (excluding cells or conditioned media). Biological samples, from which EVs were isolated for metabolomic analysis, included blood serum/plasma, urine, stool, and conditioned medium (CM) from human cell lines. In six of the included studies, tumour diagnosis was confirmed through clinically validated procedures. Five studies reported histopathological confirmation (Altadill et al., 2016; Liu et al., 2023; Puhka et al., 2017; Yang et al., 2023; Zhu et al., 2021), and one study assessed patients against the International Union Against Cancer and the American Joint Committee on Cancer in 2013 criteria (Kim et al., 2020). In two studies, patient samples were obtained from established biorepositories (Clos-Garcia et al., 2018; Soupir et al., 2023). Although biorepository use does not itself constitute a clinically validated diagnostic procedure, these studies were included because such repositories typically provide high-quality patient samples with associated clinical information, which may encompass validated diagnostic confirmation. Although one study did not include any information regarding patient diagnosis, it was retained in the review as it provided information on cancer staging (Eylem et al., 2020). All studies using human participants also included a non-cancer control group.

**Table 3:** Sample characteristics of the included studies: cancer type, sample type, and number of samples

Study	Cancer Type	Sample Type	Number of Samples
Altadill et al., 2016	Endometrioid Adenocarcinoma	Plasma	Endometrioid adenocarcinoma = 19 Healthy control = 13
	Pancreatic	Conditioned culture medium from PANC1 cells	N/A
Clos-Garcia et al., 2018	Prostate	Urine	Prostate cancer = 31 Benign Prostate Hyperplasia = 14
Čuperlović-Culf et al., 2020	Glioblastoma	Conditioned culture media from U118, LN-18, and A172 cells	N/A
Eylem et al., 2020	Colorectal	Conditioned culture media from FHC and HT-29 cells	N/A
		Serum	Colorectal cancer = 11 Healthy control = 8
Kim et al., 2020	Colorectal	Stool	Colorectal cancer = 32 Healthy control = 40
Liu et al., 2023	Prostate	Serum	Prostate cancer = 17 Castration resistant prostate cancer = 15 Healthy control = 12
Palviainen et al., 2020	Prostate	Conditioned culture media from PC-3, and PNT2 cells	N/A
	Cutaneous T-Cell Lymphoma	Conditioned culture media from Mac-2A, and PBMC cells	N/A
	Colorectal	Conditioned culture media from RKO and CCD 841 CoN cells	N/A
Puhka et al., 2017	Prostate	Urine	Prostate cancer = 3 Healthy control = 3
		Plasma	
Soupir et al., 2023	Prostate	Plasma	Prostate cancer = 10 Lung cancer = 14 Healthy control = 10
	Lung	Plasma	
(Yagin et al., 2023)	Colorectal	Stool	Colorectal cancer = 36 Healthy control = 40
Yang et al., 2023	Lung	Urine	Early lung cancer = 33 Lung cancer = 42 Healthy control = 27
Zhu et al., 2021	Oesophageal Squamous Cell Carcinoma	Plasma	Recrudescent ESCC = 34 Non-Recrudescent ESCC = 37 Health control = 20

Table 4 provides a comparison of the experimental design of each study, including the EV isolation and confirmation techniques, as well as the metabolite extraction and analysis methods employed. The predominant isolation technique utilised was ultracentrifugation. Successful isolations were verified through a variety of techniques, including nanoparticle tracking analysis, western blot for EV-associated proteins, transmission electron microscopy, electron microscopy, bicinchoninic acid assay test, and immunoblotting. It is important to note that two of the studies included in the review did not provide any details regarding the use of EV confirmation tests (Kim et al., 2020; Yagin et al., 2023). Consequently, it is difficult to determine whether they analysed EVs or other non-EV particles that have been co-isolated.

**Table 4:** A summary of the techniques used in the included literature

Technique	Reference											
	(Altadill et al., 2016)	(Clos-Garcia et al., 2018)	(Čuperlović-Culf et al., 2020)	(Eylem et al., 2020)	(Kim et al., 2020)	(Liu et al., 2023)	(Palviainen et al., 2020)	(Puhka et al., 2017)	(Soupir et al., 2023)	(Yagin et al., 2023)	(Yang et al., 2023)	(Zhu et al., 2021)
EV Isolation Technique												
Ultracentrifugation	X	X	X	X		X	X	X				
Centrifugation					X							
EXODUS device											X	X
Incubation										X		
SBI SmartSEC™ Single for EV Isolation™									X			
Takara Capturem™									X			
Fujifilm Wako MagCapture™									X			
EV Confirmation Technique												
Nanoparticle Tracking Analysis	X	X				X	X	X	X		X	X
Western Blot	X	X	X			X	X	X			X	X
Transmission Electron Microscopy				X		X					X	X
Electron Microscopy		X					X	X				
Bicinchoninic Acid Assay				X								
Immunoblotting				X								
Solvents Used for Solvent-Based Metabolite Extractions												
Methanol									X			X
Acetonitrile			X				X	X				
Acetonitrile and Methanol						X					X	
Solvents Used for Liquid–Liquid Metabolite Extractions												
Methanol and Chloroform	X	X		X								
Instrumental Technique Used for Metabolomic Analysis												
Gas Chromatography-Mass Spectroscopy (GC–MS)				X	X					X		
Liquid Chromatography-Mass Spectroscopy (LC–MS)	X	X				X	X	X	X	X	X	X
Nuclear Magnetic Resonance Spectroscopy (NMR)			X									
Metabolite Identification												
Human Metabolome Database (HMDB)	X	X	X	X			X	X				
Madison Metabolomics Consortium Database	X											
Comparison to an In-house Metabolite Library									X			
Metware Database												X
Biological Magnetic Resonance Databank			X									
EVpedia								X				
MS2 Database											X	

Before metabolomic analysis, sample preparation included metabolite extraction, most commonly utilising an acetonitrile and water extraction technique or a methanol, chloroform and water extraction technique. Mass spectrometry-based analytical analysis (LC–MS, GC–MS) was the most employed metabolomic technique in 11 of the 12 included studies. The remaining experiment applied ^1^H NMR. Finally, compound identification was achieved by database matching using the Human Metabolome Database, the Madison metabolomics consortium database, and the Metware Database. One study achieved compound identification by comparison to an in-house metabolite library.

### Identified metabolites

**Fig. 3:**
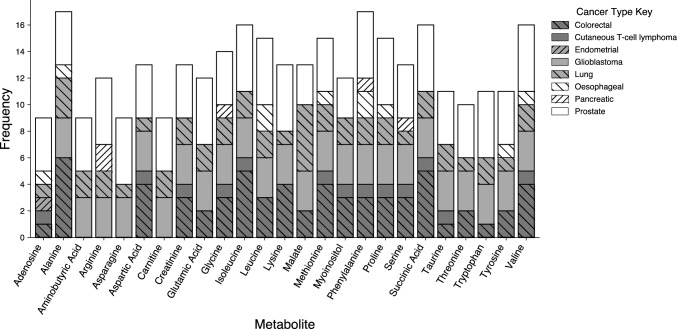
The most reported metabolites within the included articles and the cancers in which they were found. The stacked bar chart illustrates the frequency of various metabolites identified in the included articles and their association with eight types of cancer: Colorectal, Cutaneous T-cell lymphoma, Endometrial, Glioblastoma, Lung, Oesophageal, Pancreatic, and Prostate cancers

Authors reported metabolites and their differential regulation within EVs across multiple cancer types. These metabolites could help to elucidate the metabolic profiles of cancer-derived EVs and their potential biomarkers. For a comprehensive summary of the most commonly dysregulated metabolites, their associated cancer type, the biofluid they were present in, and their dysregulation patterns, see Table 5. A complete list of all identified metabolites is included in supplementary information (SI Table 1), with metabolites unique to a particular cancer type highlighted in red, potentially providing valuable information for the development of cancer-type-specific biomarker panels. Figure 3 displays the most frequently reported metabolites from the included studies and the cancer populations in which they were identified. This figure is intended as a descriptive summary of reporting trends as the included studies utilised differing EV isolation methods, biological matrices and cancer types. Consequently, the figure should be interpreted cautiously, as inter-study heterogeneity prevents direct comparison.

**Table 5:** Frequently dysregulated metabolites and their direction of dysregulation within the included studies

Cancer	CRC					Glioblastoma			PCa			CTCL	Lung	
Reference	(Eylem et al., 2020)	(Eylem et al., 2020)	(Palviainen et al., 2020)	(Kim et al., 2020)	(Yagin et al., 2023)	(Čuperlović-Culf et al., 2020)			(Palviainen et al., 2020)	(Clos-Garcia et al., 2018)	(Puhka et al., 2017)	(Palviainen et al., 2020)	(Soupir et al., 2023)	(Yang et al., 2023)
Biofluid	Serum	HT29	RKO	Stool	Stool	A172	LN18	U118	PC3	Urine	Plasma	Mac-2A	Serum	Urine
Non Essential Amino Acids (NEAAs)														
Glycine	↓	↓				↑	↑	↓	↑					
Glutamine							↓		↑		↑			
Proline	↓	↓	↑			↓	↓	↑	↑			↑	↓	↑
Carnitine						↑	↑	↓	↑	↑			↑	
Essential Amino Acids (EAAs)														
Methionine			↑			↓	↓	↓						
Phenylalanine	↓	↓							↑					
Valine	↓	↓							↑					↑
Leucine				↑	↑				↑		↑			
Isoleucine	↓	↓		↑	↑	↑	↑	↓	↑		↑			
Lysine	↓	↑		↑					↑				↓	
Tryptophan	↓	↓				↑	↑	↓	↑		↑			
Organic Acids														
Succinic Acid	↓	↓		↑	↑	↓	↓	↑	↑			↑		
Citric Acid	↓	↓				↓				↑				
Sugars														
Glucose		↑				↑	↑	↓						
Fructose	↓	↓												
Xylitol	↓	↓

### Potential biomarkers and biomarker panels

While it is important to consider metabolites that overlap between different cancers, novel diagnostic approaches should focus on cancer-type-specific biomarker panels that reflect unique pathophysiological signatures. Metabolic changes observed in EVs derived from biofluids and cell cultures highlight the potential of EV metabolomics in developing biomarker panels and targeted diagnostic tools. Such metabolic changes are denoted by (↑) for upregulated metabolites and (↓) for downregulated metabolites.

#### Colorectal cancer

Kim et al. (2020) identified two metabolic biomarkers, leucine (↑) and oxalic acid (↑), that provided a CRC predictability of 92% with 80% sensitivity and 100% specificity, respectively. However, these metabolites are not specific to CRC. For example, leucine has been reported in EVs from lung cancer, PCa, OSCC, and glioblastoma samples (Čuperlović-Culf et al., 2020; Palviainen et al., 2020; Puhka et al., 2017; Zhu et al., 2021). Additionally, oxalic acid has been detected in EVs derived from PCa and lung cancer samples (Soupir et al., 2023). To improve the accuracy of CRC diagnosis, Kim et al. (2020) propose that future studies use a combination of metabolomic and metagenomic biomarkers, with metagenomics involving the analysis of the collective genetic material of microbial communities. This combination has the potential to allow a more specific diagnosis of CRC than a single omics biomarker can provide.

Having identified the same 15 metabolites within EVs derived from the stool of CRC patients, the work by Yagin et al. (2023) supports the findings of Kim et al. (2020). In their study, Yagin et al. highlighted aminoisobutyric acid (↓), butyric acid (↓), succinic acid (↑), isoleucine (↑), and leucine (↑) as potential diagnostic biomarkers for CRC. However, contrary to Kim et al. (2020), Yagin et al. (2023) identified aminoisobutyric acid as having the highest discriminative potential and leucine as having the lowest.

In another CRC EV study, Eylem et al. (2020) identified 37 metabolites that exhibited statistically significant changes (p < 0.05) in EVs derived from CRC CM EVs and 31 from serum EVs of CRC patients. Eylem et al. (2020) reported similar dysregulation of glycine (↓), proline (↓), phenylalanine (↓), valine (↓), isoleucine (↓), tryptophan (↓), succinic acid (↓), citric acid (↓), fructose (↓), and xylitol (↓) between serum-derived and HT29 CM-derived EVs. While this study supports the conclusions of dysregulated succinic acid (↓) and isoleucine (↓), in both HT29 CM-derived EVs and serum-derived EVs, the direction of dysregulation contradicts the findings of Kim et al. (2020) and Yagin et al. (2023). Furthermore, this analysis does not identify significant dysregulation in aminoisobutyric acid, butyric acid, leucine, and oxalic acid, as reported by Yagin et al. (2023). Instead, Eylem et al. (2020) highlight monomethyl phosphate (↓) and D-pinitol (↓) as having marked alterations between EVs derived from CRC and healthy samples.

Palviainen et al. (2020) proposed that it is possible to distinguish cancer cell-derived EVs from control cell-derived EVs in *in-vitro* settings using a biomarker panel of proline (↑), succinate (↑), folate (↑), and creatinine (↑). However, their conclusion that different cancer types share a similar EV metabolome means that this biomarker panel cannot specify the exact type of cancer. While Palviainen et al. (2020) did not identify any significantly dysregulated metabolites unique to CRC, they did identify the upregulation of proline (↑) in RKO-derived EVs. Eylem et al. (2020) found similar upregulation in HT29-derived EVs but reported contrastingly downregulated proline levels in serum-derived EVs.

#### Prostate cancer

PCa-specific biomarkers found by Palviainen et al. (2020) include trimethyl-lysine (↑), isobutyryl carnitine (↑), and glycine betaine (↑). These were unique to PCa PC3-derived EVs and healthy PNT2 control-derived EVs. Palviainen et al. (2020) also reported the dysregulation of carnitine (↑) and its derivatives acetylcarnitine (↑), isobutyrylcarnitine (↑), and propionylcarnitine (↑) in EVs derived from the CM of PC3 PCa cells. Other metabolites reported as dysregulated in these EVs included glycine (↑), glutamine (↑), proline (↑), phenylalanine (↑), valine (↑), leucine (↑), tryptophan (↑), isoleucine (↑), lysine (↑), and succinic acid (↑). Palviainen et al. (2020) did not report the downregulation of any metabolites in EVs derived from CM of PC3 PCa cells.

Liu et al. (2023) concluded that exosomal metabolites have potential value as CRPC markers that facilitate the discrimination of CRPC patients from PCa patients and tumour-free controls (TFC). They analysed serum EVs from PCa, CRPC, and TFCs. From their analysis, they were able to identify two elevated metabolites, 2-(2-methylbutanoyl) (↑), and acetylglycine (↑), and three downregulated metabolites, creatinine (↓), dihydrothymine (↓), and hydroxyoctanoic acid (↓), in PCa EV samples, relative to the TFC group. Amongst the significantly dysregulated metabolites, 2-(2-methylbutanoyl), dihydrothymine, and hydroxyoctanoic acid were all unique to EVs from CRPC, PCa, and their respective controls, further supporting their potential as CRPC biomarkers. Further work by Liu et al. (2023) sought to identify differences between CRPC and PCa EVs. From this, they were able to distinguish CRPC EVs from PCa EVs, with dysregulated levels of cycloartocarpin (↑), 2-methylglutaric acid (↑), tridecanoic acid (↓), undecanoic acid (↓), and hydroxyoctanoic acid (↓) in the CRPC group.

Puhka et al. (2017) distinguished between the metabolomes of EVs derived from pre-prostatectomy, post-prostatectomy and healthy control urine samples. Their study reports dysregulated levels of leucine (↑), tryptophan (↑), adenosine (↓), glucuronate (↓), isobutyryl-L-carnitine (↓), D-ribose-5-phosphate (↓), 1-methylhistamine (↓), creatine (↓), glutathione (↓), Propionylcarnitine (↓), Isovalerylcarnitine (↓), and NAD^+^ (↓) in pre-prostatectomy patient urine EV samples as compared to EVs derived from controls and post-prostatectomy samples. Of these metabolites, glucuronate, a metabolite unique to PCa samples and their controls, displayed the most significant difference between the two groups.

In their study, Clos-Garcia et al. (2018) identified 248 metabolites, with 76 showing statistically significant differences between EVs from PCa and benign prostate hyperplasia (BPH) patients. Significantly dysregulated metabolites in PCa urine EVs included gamma-aminobutyric acid (↑), dehydroepiandrosterone sulphate (↑), arachidonic acid (↓), and phosphatidylcholine (↓). Their findings suggest that arachidonic acid levels in urine EVs have a potential role in non-invasively evaluating what is occurring in prostatic tissue in PCa. However, multivariate analysis did not show perfect separation of PCa and BPH patients. Clos-Garcia et al. (2018) additionally reported five metabolites: Cer(d18:1/16:0), Cer(d18:1/20:0), Cer(d18:1/22:0), PC(30:0), and stearoylcarnitine [AC(18:0)] with significant differences between PCa stages two and three that may allow longitudinal monitoring of PCa development.

Although the primary research focus of Soupir et al. (2023) was the comparison of EV isolation techniques, their work also compared EVs derived from CRPC, NSCLC, and healthy control samples. In their study, Soupir et al. (2023) found elevated L-cystathionine (↑) in CRPC samples compared to samples from patients with NSCLC or healthy controls. L-cystathionine was also found in EVs from the plasma and urine of PCa patients by Puhka et al. (2017) and Clos-Garcia et al. (2018). However, these two studies did not consider it a significant metabolite in distinguishing between PCa and healthy samples.

#### Lung cancer

Soupir et al. (2023) found significantly dysregulated levels of O-acetyl-L-carnitine (↑), N-alpha-acetyl-L-lysine (↓) and proline (↑) in EVs derived from NSCLC plasma samples compared to EVs derived from the plasma of healthy donor samples. Yang et al. (2023) also reported the presence of N-alpha-acetyl-L-lysine in EVs derived from lung cancer patients. However, the study did not find significant dysregulation of this metabolite.

Yang et al. (2023) additionally observed the dysregulation of proline-betaine (↑) and valine (↑) in early-stage lung cancer patients and D-proline in late-stage lung cancer patients when analysing urine-derived EVs. While these metabolites displayed significant dysregulation, they are not unique to lung cancer. Instead, Yang et al. (2023) proposed a diagnostic panel composed of kanzonol Z (↑), xanthosine, nervonyl carnitine, and 3,4-dihydroxybenzaldehyde. When applied to training, testing, and validation sets, this biomarker panel achieved an area under the curve (AUC) value of over 0.84 for distinguishing and predicting early-stage lung cancer. While xanthosine was additionally found in PCa by both Clos-Garcia et al. (2018) and Puhka et al. (2017), the remaining three metabolites were unique to lung cancer EVs.

#### Glioblastoma

Čuperlović-Culf et al. (2020) differentiated EVs derived from glioblastoma cell lines (U118, A172, and LN18) and normal human astrocyte (NHA) controls using metabolomics. Within EVs derived from A172 and LN18, there were dysregulated levels of succinic acid (↑), isoleucine (↑), glycine (↑), glucose (↑), carnitine (↑), and tryptophan (↑). Additionally, LN18-derived EVs had dysregulated glutamine (↓), threonine (↑), and homoserine (↑). Citric acid (↓) downregulation can distinguish A172-derived EVs from the other cell lines. Furthermore, Čuperlović-Culf et al*.* identified upregulation of proline (↑) in EVs derived from the CM of highly aggressive U118 glioblastoma cells. This dysregulation of proline could be utilised as a marker to differentiate U118-derived EVs from those derived from A172 cells, LN18 cells, and NHA controls. Finally, the downregulation of methionine (↓) in CM EVs from U118, A172, and LN18 cells allowed their distinction from non-cancerous NHA control-derived EVs. Čuperlović-Culf et al. (2020) identified several significantly dysregulated metabolites, but they were not specific to glioblastoma and, therefore, have limited individual applications. While Čuperlović-Culf et al. (2020) did not propose a biomarker panel, they suggested that it is possible to establish an appropriate biomarker panel for metabolically active systems. However, they conclude that this is only a possibility in the context of further research regarding biofluids (blood or cerebrospinal fluid) and sample collection procedures.

#### Oesophageal squamous cell carcinoma

While Zhu et al. (2021) do not describe the direction of dysregulation, they propose a biomarker panel made of 3’-UMP, palmitoleic acid, palmitaldehyde, and isobutyl decanoate, which can resolve recrudescent and non-recrudescent OSCC patients with an AUC of ~ 98%. The study by Zhu et al. (2021) identified 3’-UMP, a metabolite unique to OSCC and its controls, as the most significant for classifying the groups.

#### Pancreatic cancer and endometrioid adenocarcinoma

Altadill et al. (2016) looked at the effect of TGF-b treatment on the metabolite profile of PANC1-CM EVs and controls. Identified by principal component analysis (PCA), they report a clear separation of the ELVs with dysregulation of UDP-D-glucosamine (↑) and DG(20:2) /18:1/0:0 (↑) after the TGF-b treatment. Furthermore, the study was able to visually distinguish the metabolome of plasma EVs from controls and endometrioid adenocarcinoma patients using a heat map and PCA plot; however, several metabolites did not yield an accurate mass-based putative identification. While Altadill et al. (2016) do not report the identification of the significant metabolites from the EVs of endometrioid adenocarcinoma patients, they conclude that EV metabolomics has a high clinical and translational relevance for biomarker discovery.

#### Results summary

Table 6 below summarises the included studies and whether they were able to distinguish between EVs derived from patients with cancer, or cancerous cell lines and their respective healthy controls using metabolomics.

**Table 6:** Discrimination of cancer and control samples using EV metabolomic profiles

Study	Cancer TYPE	Sample type	Was differentiation achieved?
Altadill et al., 2016	Endometrioid Adenocarcinoma	Plasma	Using PCA, they were able to differentiate metabolites found in the EVs isolated from the plasma of patients with Endometrioid Adenocarcinoma compared to the controls
Clos-Garcia et al., 2018	PCa	Urine	PCA did not show a perfect separation between EVs derived from patients with prostate cancer and those with benign prostate hyperplasia
Čuperlović-Culf et al., 2020	Glioblastoma	U118, LN-18, and A172 CM	t-SNE based on EV metabolome data was able to show discrimination of U118 from LN18 and A172 cm derived EVs
Eylem et al., 2020	CRC	FHC and HT-29 CM	PLS-DA was able to distinguish between the metabolome of CRC and Healthy Control serum derived EV samples and FHC and HT-29 CM derived exosomes
		Serum	
Kim et al., 2020	CRC	Stool	Using PCA they were able to differentiate EVs derived from CRC patients from healthy controls
Liu et al., 2023	PCa	Serum	EV metabolites, elevated 2-(2-methylbutanoyl) and acetylglycine, could differentiate between PCa, CRPC, and tumor-free controls
Palviainen et al., 2020	PCa	PC3 and PNT2 CM	PCA revealed a clear separation between PC-3 and PNT2 cell line-derived EVs
	CTCL	Mac-2A and PBMC CM	Further testing with Mac-2A, PBMC, RKO and CCD 841 CoN CM they developed a biomarker panel of proline, succinate, folate, and creatinine to distinguished cancer cell-derived EVs from control cell-derived EVs in *in-vitro* settings
	CRC	RKO and CCD 841 CoN CM	
Puhka et al., 2017	PCa	Urine	While the authors do not explicitly state if they were able to distinguish between the sample types, they report that four metabolites (adenosine, glucuronate, isobutyryl-L-carnitine, and D-ribose 5-phosphate) had lower levels in the patient uEV samples before prostatectomy than in the control uEV samples and after prostatectomy uEV samples
		Plasma	
Soupir et al., 2023	PCa	Plasma	This study identified significant differences in exosome metabolic profiles in plasma exosomes isolated using SEC between CRPC and NSCLC and healthy controls
	Lung	Plasma	
Yagin et al., 2023	CRC	Stool	ROC analysis showed that five candidate biomarkers (aminoisobutyric acid, butyric acid, succinic acid, isoleucine, and leucine) can discriminate between EVs derived from CRC patients and healthy controls
Yang et al., 2023	Lung	Urine	Using a diagnostic panel composed of Kanzonol Z, Xanthosine, Nervonyl carnitine, and 3,4-Dihydroxybenzaldehyde they were able to discriminate between EVs derived from patients with lung cancer, early lung cancer and healthy controls
Zhu et al., 2021	OSCC	Plasma	Discriminated recrudescent and non-recrudescent OSCC patients using a biomarker panel made of 3’-UMP, palmitoleic acid, palmitaldehyde, and isobutyl decanoate

## Discussion

This systematic review evaluated the application of metabolomics and metabolite analysis for identifying dysregulated metabolic profiles in EVs. The secondary aim was to identify metabolites that differentiate EVs derived from cancer patients from those of healthy controls, as well as EVs derived from the conditioned media of cancer cell lines from those of corresponding healthy cell lines. Despite notable variations in EV isolation, sample preparation and metabolomic analysis techniques in the included studies, all the studies distinguished between EVs derived from patients with cancer, or cancerous cell lines and their respective healthy controls using metabolomics.

The dysregulation of cellular metabolism to support continuous cell growth and proliferation is a recognised hallmark of cancer (Hanahan & Weinberg, 2011). Where it is feasible to identify metabolites and their derivatives unique to cancerous cells, their presence in biological fluids has the potential to serve as a unique biomarker or part of a biomarker panel (Hanahan & Weinberg, 2011; Pavlova & Thompson, 2016). The metabolites identified from this review provide a summary of the metabolic signatures arising from EVs derived from patients with cancer or conditioned medium from cancerous cell lines. Notably, AAs, including alanine, phenylalanine, valine, and isoleucine, emerged as the most frequently reported metabolite type with significant dysregulation between EVs derived from patients with cancer, or cancerous cell lines and their respective healthy controls. This observation indicates the crucial role amino acid metabolism plays in differentiating these EV populations. Within cancerous cells, metabolic reprogramming of AAs is intrinsically linked to the synthesis of proteins and nucleotides, modulation of signalling pathways such as mTOR, regulation of tumour metabolism, and epigenetic modifications (Faubert et al., 2020). This AA dysregulation is reflected in EVs, which not only reflect the altered metabolic state of their cancerous cell of origin but also participate in EV-mediated transfer of AAs from donor cells to recipient cells (Hirosawa et al., 2025). This cellular communication facilitated by EVs has a multifaceted effect on the tumour microenvironment and intercellular communication, promoting processes such as metastasis, angiogenesis, and immune evasion (Liu et al., 2024). This dysregulation of AAs, amongst other metabolite dysregulations, can be leveraged to develop biomarker panels for distinguishing EVs derived from cancerous versus non-cancerous cells.

While there are many potential reasons for the dysregulation of cellular metabolism, one such explanation could be the Warburg effect, a hallmark of most cancers, which sees cancerous cells favouring glycolysis over oxidative phosphorylation even in the presence of oxygen (Hanahan & Weinberg, 2011; Warburg, 1956). This increased glycolysis facilitates the biosynthesis of nucleotides and AAs needed for cell assembly and growth (Pavlova et al., 2022). Additionally, it causes an increase in glucose consumption and lactate production (Heiden et al., 2009). Other dysregulated metabolites, such as organic acids, including citrate and succinate, act as intermediaries for the TCA cycle (Heiden et al., 2009). These intermediaries are often precursors to fatty acids and nucleotides, which act as building blocks for macromolecules such as DNA, proteins, and lipids (Heiden et al., 2009). However, it is worth noting that the metabolites mentioned above could also exhibit significant dysregulation in other diseases. For example, there is dysregulation of citrate levels in the prefrontal cortex of patients with Alzheimer’s disease (Paglia et al., 2016). Valine levels are significantly higher in patients with acute heart failure (Klobučar et al., 2023). Finally, phenylketonuria causes upregulation of phenylalanine, valine, glycine and methionine in patient samples (Cannet et al., 2023). Despite the challenges posed by their significance in multiple diseases, these metabolites provide valuable insights into the dysregulation of underlying cellular processes. This shared dysregulation between cancer and other diseases can reveal common mechanisms of disease progression, offering potential therapeutic targets.

While this review demonstrates the progress made in identifying potential cancer biomarkers through EV metabolomics, the challenge remains in developing and validating comprehensive metabolite biomarker panels to detect specific cancer types. Such biomarker panels have the potential to offer a more robust and powerful separation than single biomarkers (Smith et al., 2021). However, before clinical implementation, these panels must undergo validation across large and diverse patient populations. Such validation will enable the demonstration of statistically significant biomarkers and their applicability across populations (Broadhurst & Kell, 2006; Hwang & Weiss, 2016). The sample sizes of the included studies are relatively small, ranging from 6 to 102 samples and therefore lack statistical confidence (Dunn et al., 2011). In metabolomic phenotyping studies, free-to-use statistical tools such as the Data-driven Sample Size Determination algorithm and MetSizeR can be used during experimental design for sample size determination, as done in the study by Yagin et al. (2023) (Billoir et al., 2015). The utilisation of such tools can ensure adequate sample sizes are utilised to provide appropriate statistical power for biomarker validation (Billoir et al., 2015).

In addition to the main finding of dysregulated metabolites between EVs derived from patients with cancer, or cancerous cell lines and their respective healthy controls, the reviewed literature also highlights the lack of consistency in reported metabolites for different cancer types. In some cases, there is contradictory metabolite dysregulation between studies investigating the same cancer type using different sample types. Discrepancies between reported metabolite regulation could be due to the heterogeneity of cancer types, stages, and individual patient variations. Moreover, alterations to the metabolome could also arise from inherent differences in the samples, as EVs were isolated from various sources such as blood plasma, blood serum, urine, stool, and CM across the studies. Cell models are invaluable preclinical models due to their easy management, cost-effectiveness, immortality, restricted cellular heterogeneity, and rapid proliferation rates, but their growth conditions differ from the in vivo environment (Idrisova et al., 2023; Sajjad et al., 2021). The effect of these differences can be seen within the included studies, whereby analysis of samples from cell models and biological fluids yielded different dysregulation of metabolites. An example of this is the downregulation of proline in EVs derived from the serum of CRC patients found by Eylem et al. (2020) and the contrasting upregulation identified in EVs derived from CM of RKO and HT29 CRC cell lines found by Palviainen et al. (2020).

These biospecimen-related differences were not limited to CM, with further differences identified between biological fluid types. For example, CRC EVs from patients’ stool displayed isoleucine upregulation, but serum EVs showed downregulation (Eylem et al., 2020; Kim et al., 2020; Yagin et al., 2023). Metabolomic differences arising in EVs isolated from blood-derived biofluids could be affected by elements of sample collection, including the type of collection tube used. Six studies analysed EVs derived from blood serum or blood plasma. Of these, two studies used blood collection tubes coated with Ethylenediaminetetraacetic Acid (EDTA) (Altadill et al., 2016; Soupir et al., 2023). The remaining four studies did not disclose the type of blood collection tube used (Eylem et al., 2020; Liu et al., 2023; Puhka et al., 2017; Zhu et al., 2021). For serum collection, tubes often contain a polymeric film or silicone coating on the inner tube wall to induce coagulation (López-Bascón et al., 2016). However, these coatings can impact subsequent analysis and metabolism within the sample. Work by López-Bascón et al., (2016) analysing polymeric gel-coated serum tubes, found changes in serum metabolism of AAs such as alanine, proline, and threonine. They also found changes in the levels of glycerol and lactic acid. Often, tubes have anticoagulant coatings such as EDTA or heparin for blood plasma collection (López-Bascón et al., 2016). However, EDTA can lead to interference and matrix effects in MS-based techniques (Barri & Dragsted, 2013). EDTA in plasma potentially induces ion suppression or enhancement of polar metabolites co-eluting with EDTA peaks (Barri & Dragsted, 2013). Additionally, intrinsic factors such as a patient's age, race, sex, diet, and lifestyle can cause changes in the characteristics, concentrations, and metabolite regulation of EVs (Noren Hooten et al., 2022). However, during the design of metabolomic studies, mitigations can be made to minimise the impact of these factors. Investigators match samples and healthy controls as closely as possible to account for effects from age, sex, ethnicity, and lifestyle (Eylem et al., 2020; Kim et al., 2020; Liu et al., 2023; Soupir et al., 2023; Yang et al., 2023). Additionally, where appropriate, patients are asked to be in a fasted state before sample collection to minimise the effect of diet on the metabolome.

When evaluating the clinical potential of EVs, it is important to consider the added value of EV metabolomics relative to direct profiling of whole biofluids. Comparative studies indicate only partial overlap between EVs and their source matrices, suggesting that analysis of EVs has the potential to provide a source of complementary low-abundance metabolites. When analysing urinary EVs (uEVs), Puhka et al. (2017) reported that fewer than half (43%) of the metabolites detected in control urine were consistently present in matched vesicle fractions, yet six metabolites, including γ-glutamylcysteine, were reliably detected only in uEVs, highlighting their capacity to capture low-abundance compounds otherwise undetectable in bulk urine. In the same study, platelet EVs were found to share the majority of metabolites (92%) with platelets but also contained 11 additional metabolites that were below the limit of detection in platelet samples (Puhka et al., 2017). Similarly, Altadill et al. (2016) found little overlap between the metabolites identified in EV fractions from plasma and regular plasma profiling, which they attribute to the sensitivity range of their instrument. Together, these findings highlight that while metabolomic analysis of biofluids provides broad coverage, EVs can be rich in low-abundance metabolites that would be otherwise undetected in bulk biofluid analysis.

EV yield, composition, and heterogeneity are impacted both by pre-analytical processing procedures and their source of origin, such as biological fluids, tissues, and conditioned cell culture media (Dudzik et al., 2021). Consequently, EV source and isolation techniques should be selected to ensure compatibility with downstream applications. Ultracentrifugation, the most frequently used approach within the included studies, can range from high to low yield and purity and has variable reproducibility, due to optimisation of the starting sample, rotor type, and the g-forces applied (Clos-Sansalvador et al., 2022; Welsh et al., 2024). This limits its cross-laboratory comparability. Size-exclusion chromatography (e.g., EXODUS device) provides high EV purification and ease of use. However, the levels of co-isolates present in the resulting samples depend on the type of starting sample, the stringency of fractionation, and the stationary phase used (Clos-Sansalvador et al., 2022). Furthermore, commercially available immunoaffinity-based kits (e.g., SBI SmartSEC™, Takara Capturem™, Fujifilm Wako MagCapture™) improve specificity for EV subtypes and are compatible with downstream analyses, though they may require specialised equipment and reduce scalability for clinical translation. Separation using these kits can be limited by the binding capacity of the beads, potentially leading to low yield and reduced ability to detach the functional product of interest for downstream applications (Clos-Sansalvador et al., 2022). In addition, EV quality and quantity are impacted by pre-analytical variables, including sample stability, storage conditions, and freeze–thaw cycles. During experimental design, storage parameters such as duration, temperature and the use of protective additives or storage solutions must be considered, and freeze–thaw cycles must be minimised as repeated thawing can compromise EV morphology, function, and cargo integrity (Ahmadian et al., 2024; Dudzik et al., 2021).

Untargeted metabolomics of EVs requires metabolite extraction to ensure the release of all metabolites from within the lipid membrane of the EV structure, a process complicated by the chemical diversity and dynamic range of metabolites found with EVs (Dudzik et al., 2021). Furthermore, there is evidence to suggest that the biological interpretation of metabolomics data can be impacted by the choice of metabolite extraction protocol, particularly by the polarity of the solvents used in solvent-based or liquid–liquid extractions (Duportet et al., 2012). While these extraction approaches enable metabolite recovery, their lack of specificity can lead to complex mixtures that hinder the detection of low-abundance metabolites (Lepoittevin et al., 2023). Subsequently, when designing a metabolite extraction protocol, metabolites of interest, metabolite concentrations and compatibility with downstream analyses should be considered (Dudzik et al., 2021).

Downstream analytical platforms can include GC–MS, LC–MS, and ^1^H-NMR. Each method offers different sensitivity, resolution, and limits of detection and quantification, which influence the subset of metabolites that can be detected within EVs (Palomo et al., 2014). Analytical method selection and optimisation are particularly critical in EV metabolomics, where metabolite abundance is often low and sample volume is limited (Ramirez et al., 2018). Optimisation of the analytical conditions, such as column selection in LC–MS, metabolite derivatisation in GC–MS and pulse sequence parameters in NMR, can influence metabolite resolution, identification and quantification (Geller et al., 2021; Singh et al., 2023). Collectively, these methodological factors complicate inter-study comparison and limit the interpretability of current EV metabolomics findings. Standardised optimisation strategies and transparent reporting of analytical procedures are therefore essential to ensure reproducibility and advance the development of robust EV-derived metabolite biomarkers.

Finally, the dynamic nature of EV cargo necessitates a comprehensive understanding of how different factors, such as isolation, storage conditions, metabolite extraction, and metabolomic methods, may influence the results. In recent years, the EV research community has recognised the need for standardised methodological approaches for EV isolation, characterisation, and functional analysis. This standardisation is crucial for ensuring the reproducibility and reliability of results across different studies and facilitating inter-study comparison. To address these issues, initiatives such as Minimal Information for Studies of Extracellular Vesicles (MISEV) guidelines have been developed by the International Society of Extracellular Vesicles (ISEV) (Lötvall et al., 2014; Théry et al., 2018; Welsh et al., 2024). The MISEV guidelines aim to outline experimental and reporting requirements specific to the EV field. These requirements include precise reporting of methods, the use of sufficient controls, and the need for EV characterisation by multiple complementary techniques to ensure biomarkers or functions are appropriately associated with EVs and not with other co-isolated materials (Lötvall et al., 2014; Théry et al., 2018; Welsh et al., 2024). In parallel, EV-TRACK has been developed as a centralised, crowdsourced knowledge database in which authors can report EV biology and methodology as per MISEV guidelines (Van Deun et al., 2017). However, due to the limited available research on EV metabolomics, there are no specific guidelines for reporting EV metabolomic studies. EV metabolomics guidelines could be created by adapting existing guidelines on metabolomics reporting, such as those outlined by the Metabolomics Standards Initiative (MSI) or EV analysis reporting in other fields (Salek et al., 2013; Sansone et al., 2007). Such guidelines for metabolomic analysis of EVs could facilitate data transparency and study reproducibility, especially where reporting of the identification and direction of metabolite dysregulation is concerned.

## Conclusions

This systematic review analysed current findings on the metabolomic analysis of EVs and highlighted the specific metabolites that distinguish EVs from biofluids obtained from patients with cancer or conditioned cancer cell media and their respective controls. Despite methodological variations across the 12 included studies, distinct EV metabolic signatures were consistently reported, providing an emerging avenue for cancer biomarker discovery. While amino acids such as alanine, phenylalanine, valine, and isoleucine emerged as the most frequent metabolites to be identified as dysregulated, metabolites only identified as dysregulated in one cancer type, such as glucuronate in PCa, nervonyl carnitine in lung cancer, and 3’-UMP in OSCC, offer potentially valuable insights for the development of candidate cancer-type–specific biomarker panels. However, the findings reported in this review remain preliminary, and further studies need to be conducted to assess the clinical utility of such biomarker panels and validate them in larger and clinically diverse cohorts.

Interpretation of the literature was limited by the heterogeneity of cancer types, sample origin, EV characterisation, isolation techniques, and metabolomic platforms utilised by the included studies, which complicated inter-study comparison and weakened the strength of current evidence. To address this, the field of EV metabolomics must take steps to enhance the reproducibility of results through the adoption of standardised guidelines for EV characterisation, metabolite identification, and results reporting. Consequently, future experimental studies should integrate robust validation of EV isolation and characterisation, adhere to emerging guidelines such as MISEV and ensure the implementation of transparent methodological and data reporting practices to strengthen the reliability of EV-based metabolite biomarker discovery.

## Supplementary Information

Below is the link to the electronic supplementary material.

Supplementary file1 (XLSX 48 KB)

## Data Availability

No datasets were generated or analysed during the current study.
